# Supplementary material for: Parkinson’s paradox: alpha-synuclein’s selective strike on SNc dopamine neurons over VTA
Source: NPJ Parkinsons Dis. 2025 Jul 11;11:207. doi: 10.1038/s41531-025-01055-3 (PMC12254511; doi:10.1038/s41531-025-01055-3)
Supplement: Supplementary file 1 — Supplemental Figures. [file 41531_2025_1055_MOESM1_ESM.pdf]

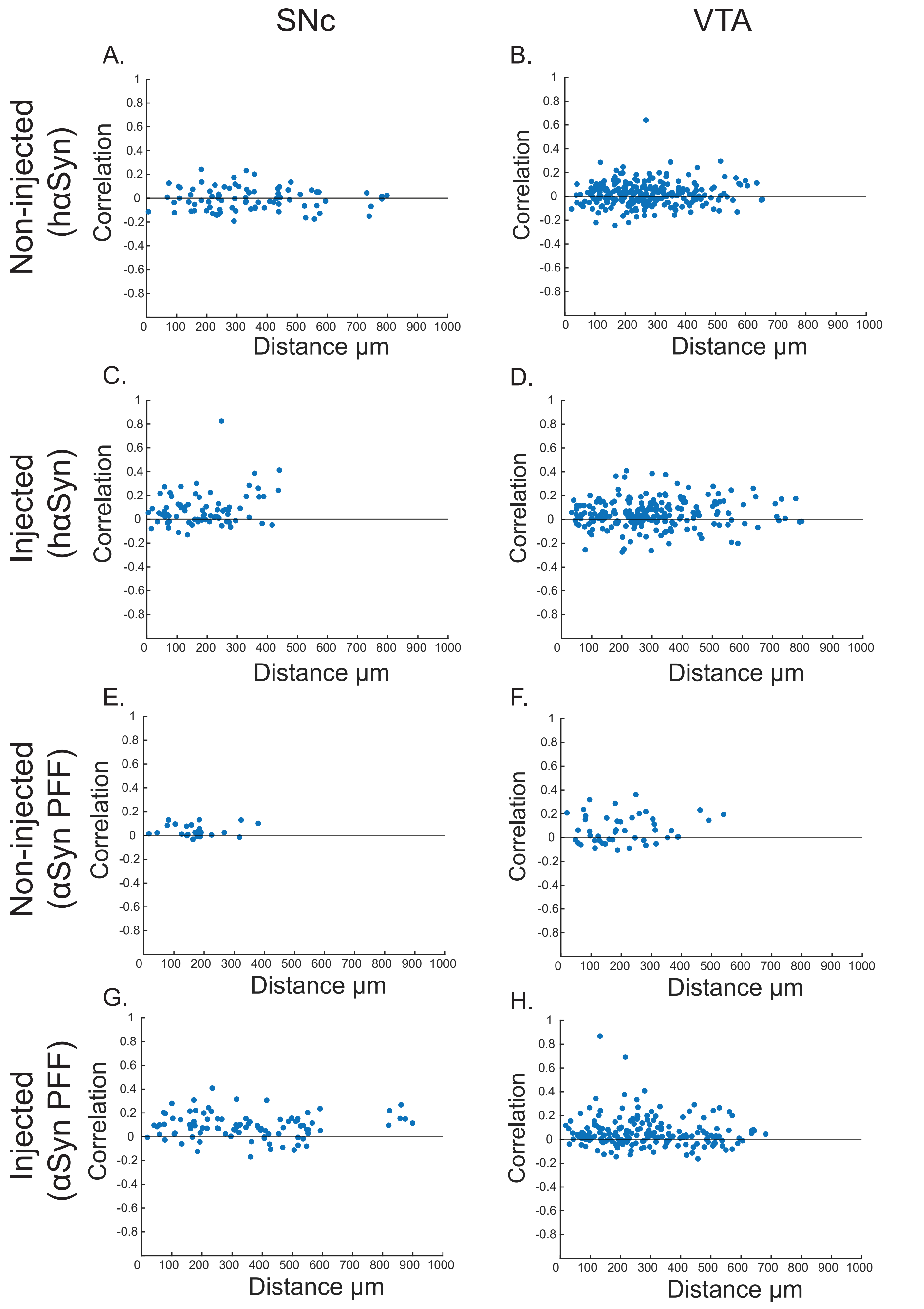

Supplemental Figure 1. Neuronal correlation activity does not correlate with inter-cell distance. Plots illustrate the relationship between correlation values and inter-neuron distance in the SNc for the non-injected hαSyn hemisphere (A), injected hαSyn (C), non-injected αSyn PFF hemisphere (E), and injected αSyn PFF hemisphere (G). Corresponding plots for the VTA are shown for the non-injected hα Syn hemisphere (B), injected hαSyn (D), non-injected αSyn PFF hemisphere (F), and injected αSyn PFF hemisphere (H).

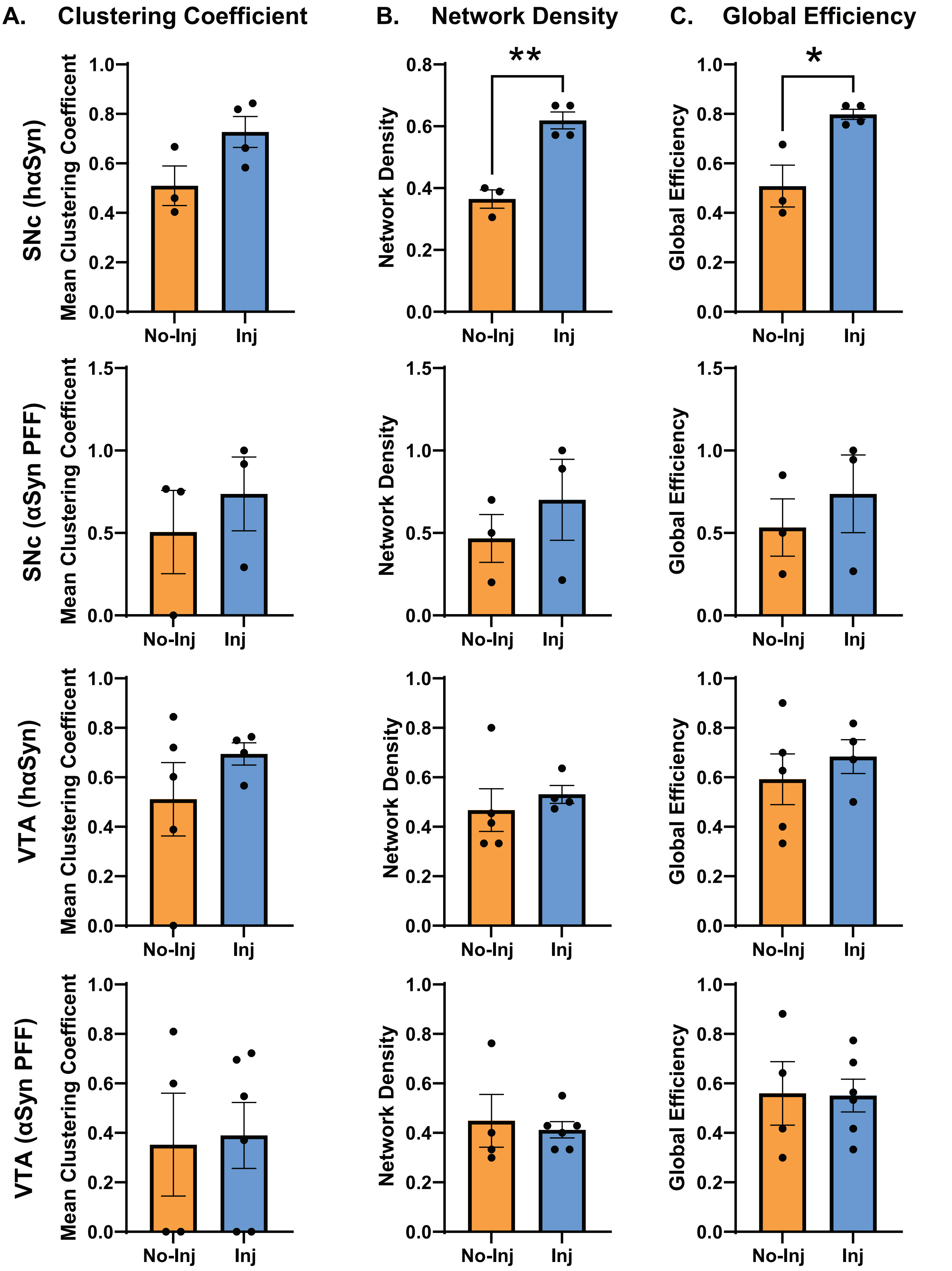

Supplemental Figure 2. hαSyn increases global network indices but not subnetwork parameters. (A) Mean clustering coefficient in hαSyn- or αSyn PFF-injected, compared to the non-injected hemisphere. (B) Network density in hαSyn- or αSyn PFF-injected, compared to the non-injected hemisphere in the SNc and VTA. (C) Global efficiency in hαSyn- or αSyn PFF-injected, compared to the non-injected hemisphere in the SNc and VTA (n = 3-5 independent biological replicates, t-test, * $p<0.05$, ** $p<0.01$).
